# Seasonal Drivers of Density in a Subarctic Population of Northern Red‐Backed Voles

**DOI:** 10.1002/ece3.73142

**Published:** 2026-02-19

**Authors:** Sarah Swanson, Melanie Flamme, Josh Schmidt, Shawn Crimmins, Carl Roland, Knut Kielland

**Affiliations:** ^1^ Department of Biology and Wildlife University of Alaska Fairbanks Fairbanks Alaska USA; ^2^ U.S. National Park Service Yukon‐Charley Rivers National Preserve and Gates of the Arctic National Park and Preserve Fairbanks Alaska USA; ^3^ U.S. National Park Service Central Alaska Network Fairbanks Alaska USA; ^4^ U.S. Geological Survey, Indiana Cooperative Fish and Wildlife Research Unit Purdue University West Lafayette Indiana USA; ^5^ Institute of Arctic Biology University of Alaska Fairbanks Fairbanks Alaska USA; ^6^ U.S. National Park Service Denali National Park and Preserve Fairbanks Alaska USA

**Keywords:** boreal forest, *Clethrionomys rutilus*, cyclicity, density dependence, northern red‐backed vole, spruce mast

## Abstract

Northern red‐backed voles ( *Clethrionomys rutilus* ) are an important species in the boreal forest ecosystem, both as herbivores and as a key food source for many mammalian and avian predators. They exhibit dramatic inter‐ and intra‐annual population fluctuations, for which causes are not entirely known. We monitored northern red‐backed vole densities in Denali National Park and Preserve through time with the goal of examining how environmental factors influenced density over time. Using a 30‐year record of mark‐recapture data, we used spatially explicit capture‐recapture methods to estimate autumn and early summer densities each year. We assessed cyclic patterns in density, variation in amplitude, and any periodicity of population fluctuations using post hoc linear modeling. We found that the vole population appeared to be cyclic with a 2–4 year period, although the pattern varied somewhat among sampling sites. Our results indicated an association between white spruce ( *Picea glauca* ) seed production and vole density, implying white spruce seeds were either an important source of food during winter seasons, or that the environmental triggers that promote high seed fall were also associated with increased vole density. We also found a negative effect of an autumn harshness index, indicating winter conditions play a role in vole density in the following season. Finally, we found evidence of a negative density‐dependent relationship between autumn and early summer. Together, these findings suggest a system in which density dependence and cyclic relationships are irregular but highly influential, with environmental effects capable of enhancing or moderating their impact. Continued monitoring of voles, alongside more thorough assessments of environmental conditions, may provide additional insight into the complex population dynamics of this species.

## Graphical abstract

In this study, we analyzed 30 years of northern red‐backed vole population data from Denali National Park to examine how environmental factors influenced density over time. We found that this population showed cyclic population fluctuations with a 2–4 year period, with some variation between sampling sites, and that densities were positively associated with white spruce seed production and negatively influenced by harsh autumn conditions. This, coupled with evidence of density dependence between autumn and early summer, suggests a system in which density dependence and cyclic relationships are irregular but highly influential, with environmental effects capable of enhancing or moderating their impact.

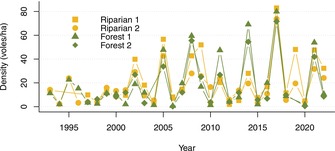

## 1 Introduction

Small mammals serve as both a vital prey base and prominent herbivores in boreal ecosystems, and can thus have a substantial impact on trophic dynamics year‐round (Gilg et al. 2003, 2009; Hanski et al. 2001; Ims and Fuglei 2005). However, small mammals can be a wildly fluctuating food source, with populations varying dramatically both within and among years (Boonstra and Krebs 2012; Cornulier et al. 2013; Fauteux et al. 2021; Mihok et al. 2019). These population fluctuations directly affect a variety of avian and mammalian predators, so understanding these trends is important for understanding food webs in boreal regions (Ims and Fuglei 2005).

Factors influencing small mammal populations include both physical and biological, the relative importance of which have been a topic of debate for decades. In some geographic areas, voles and lemmings exhibit cyclic populations that peak every 3–5 years (Fauteux et al. 2015; Krebs and Myers 1974; Stenseth 1999), while populations in other areas show patterns that are muted (Boonstra and Krebs 2012) or may be disappearing due to climate change (Ims et al. 2008; Cornulier et al. 2013, but see Korpela et al. 2013). Drivers of these cyclic fluctuations are not well understood, but the most common explanations are predation (Hanski et al. 2001; Klemola et al. 2002; Korpimäki et al. 2002) or maternal effects (Boonstra, Krebs, and Stenseth 1998; Ginzburg and Krebs 2015). The latter can take the form of altered growth rate (animals born during high‐density years grow more quickly than those born during lower‐density years) or reproductive capacity, indicated by prolonged stress responses affecting body mass and reproductive hormone response (Boonstra, Hik, et al. 1998; Sundell et al. 2019). Amplitudes of these cycles (or abundances for non‐cyclic populations) appear to be influenced by numerous factors, including food availability (Elias et al. 2006; Krebs et al. 2010; Johnsen et al. 2017; Schmidt et al. 2018), predator interactions (Ginzburg and Krebs 2015), and climate (Bilodeau et al. 2013; Domine et al. 2018; Gilg et al. 2009; Korslund and Steen 2006). Climate change is occurring at a rapid rate in North American boreal forest regions (Overland et al. 2019), and the effects on the cyclic dynamics of small mammals have remained largely unstudied. Thus, understanding the direct and indirect effects of climate change on small mammals in boreal systems is a critical aspect of predicting large‐scale ecosystem effects.

Prominent signs of climate change in interior Alaska are altered winter conditions, including higher temperatures, shorter snow season, and more variable snow‐fall (Lader et al. 2020; Littell et al. 2018; Overland et al. 2019). Changing winter conditions are known to disrupt multiple trophic levels within high‐latitude ecosystems (Penczykowski et al. 2017; Terraube et al. 2015) and may be particularly significant for small mammals. Overwinter survival is a limiting factor for northern red‐backed vole populations, for which a decrease of 50% or more during the colder months is not uncommon (Boonstra and Krebs 2012). Snow conditions directly affect small mammal survival by influencing capacity to escape predators (Lindström and Hörnfeldt 1994), find food (Gilg et al. 2009; Korslund and Steen 2006), and avoid thermal stress, especially hypothermia (Bilodeau et al. 2013). To fulfill these needs, snow must be sufficiently deep to provide insulation (Duchesne et al. 2011), of low density near the ground to ensure mobility and food accessibility, and long‐lasting enough to offer the previous benefits for the duration of winter (Bilodeau et al. 2013).

Other snow‐related factors influencing small mammal survival include rain‐on‐snow events, which have been predicted to become more frequent in Alaska under most climate change scenarios (Rennert et al. 2009). Such events may limit small mammal mobility and food accessibility by fragmenting the subnivean space, increasing snow density, and coating food sources in ice (Berteaux et al. 2017; Bilodeau et al. 2013; Ims and Fuglei 2005; Soininen et al. 2015). This could also increase metabolic demands by reducing the insulative value of the snowpack and possibly even their own fur if unable to stay dry (Berteaux et al. 2017). A more thorough analysis of winter conditions and their ramifications for small mammals would aid in predicting how their populations may respond to more varied winter weather. However, assessing the impacts of such conditions on small mammal population dynamics is challenging due to the data needs and complexities associated with understanding the drivers of cyclical populations.

To address this issue, we analyzed 30 years of northern red‐backed vole population data from Denali National Park and Preserve. Previous work suggested that northern red‐backed voles (hereafter referred to as ‘voles’, unless another species is specified) in this area retained cyclical dynamics as recently as 2014 (Schmidt et al. 2018), although recent observations suggested the dynamics of voles in this region may have been disrupted by recent anomalous weather and winter conditions (Overland et al. 2019). We hypothesized that parameters such as low summer primary productivity, warm weather events during winter, limited snowfall, and shorter snow season could negatively impact vole populations by reducing abundance. In addition, we included data on white spruce ( *Picea glauca* ) seed abundance from a nearby area to assess impacts of a potential food source, predicting that high levels of seed availability would be linked to increases in density the following year. Lastly, we used density estimates from autumn and the following early summer to explore climate factors affecting winter population dynamics, from which we predicted that late snowfall and ice layers in the snowpack would be followed by low early summer vole population densities. Our exploration of the roles of weather conditions, primary productivity, and density dependence on vole dynamics will help to increase our collective understanding of population cycles in small mammals in the subarctic.

## 2 Methods and Materials

### 2.1 Study Site

**FIGURE 1:**
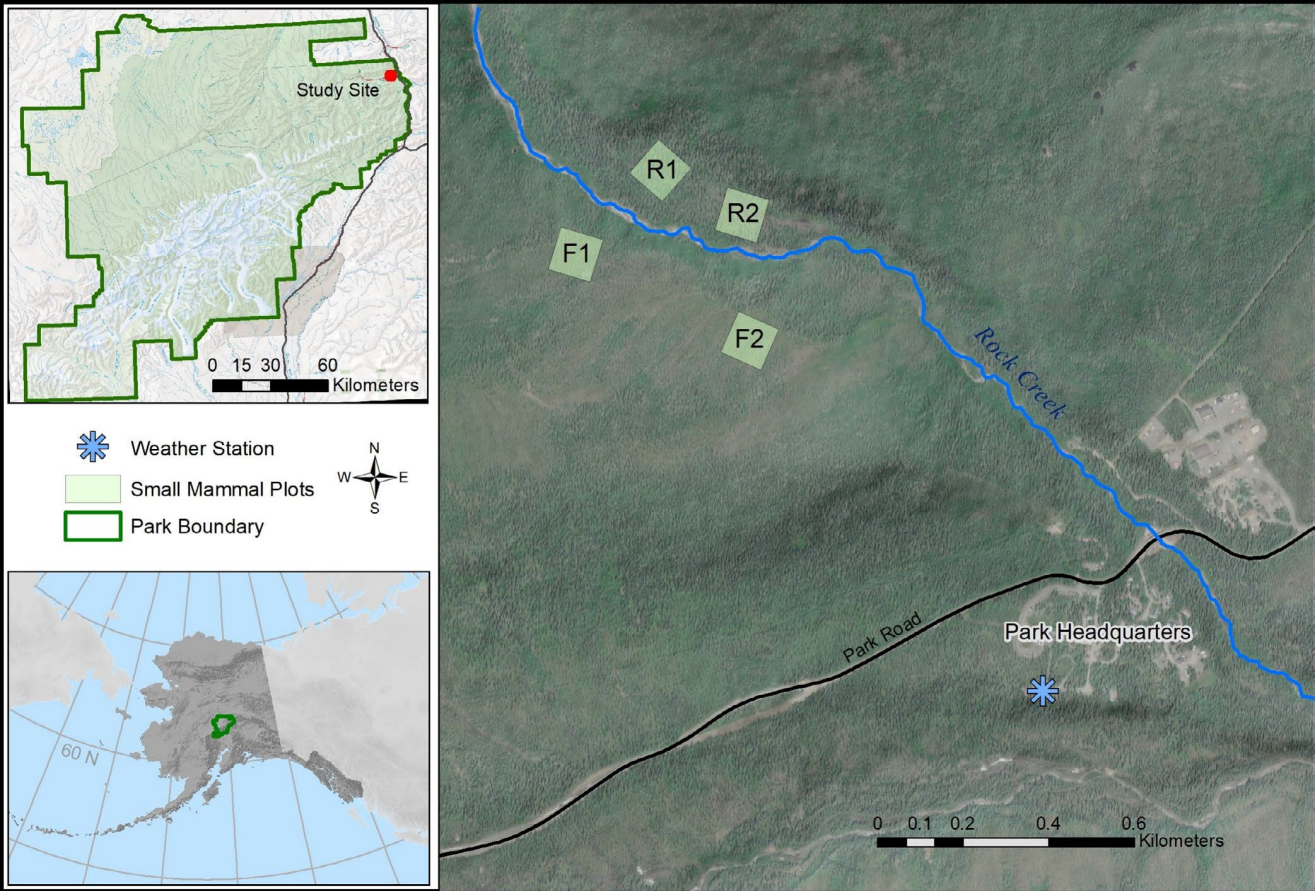
Location of the small mammal monitoring plots in Denali National Park and Preserve. Forest and riparian grids are located on opposite sides of Rock Creek (shown by the blue line). The weather station is marked with the blue asterisk.

**FIGURE 2:**
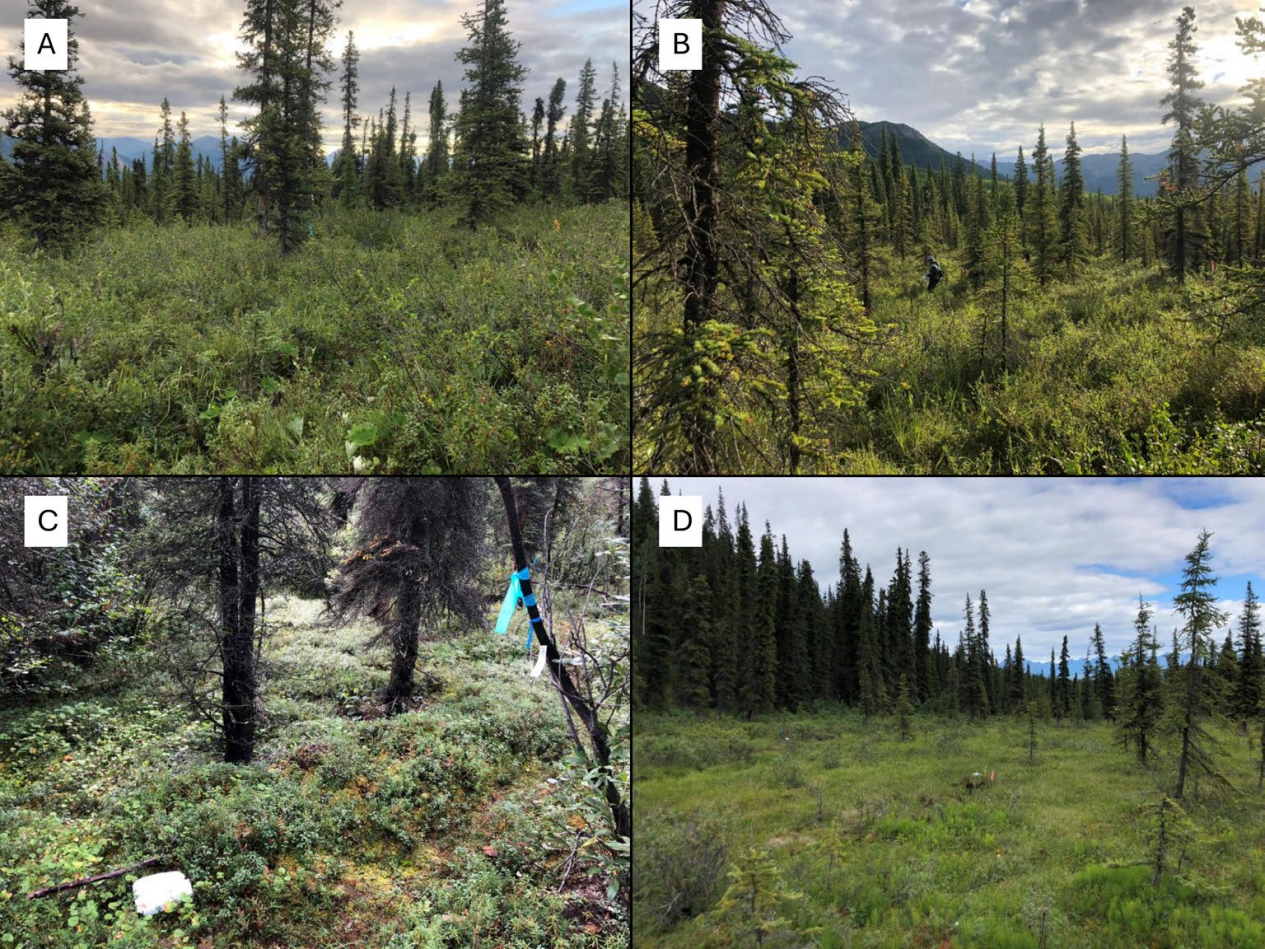
Photographs showing vegetation and structure of the four plots included in this study. (A) Forest 1 (F1), (B) Forest 2 (F2), (C) Riparian 1 (R1). (D) Meadow on bottom half of Riparian 2 (R2). Photographs by S. Swanson.

Our study area was located in the Rock Creek drainage of Alaska's Denali National Park and Preserve, at approximately 735 m elevation (Figure 1). The area had a continental climate characterized by short, warm summers and long, cold winters. We sampled small mammals at four 100 × 100 m plots in the Rock Creek drainage, two in a riparian area and two on an adjacent forested ridge (Figure 1). The forest plots were located in an area of sparse white spruce ( *P. glauca* ) and occasional black spruce ( *P. mariana* ). Trees were underlain with mosses, Bigelow's sedge ( *Carex bigelowii* ), blueberries ( *Vaccinium uliginosum* ), shrub birch ( *Betula nana* ), low‐bush cranberries ( *V. vitis‐idaea* ), Labrador tea ( *Rhododendron groenlandicum* ), and other small shrubs (Figure 2A,B). Riparian plots contained a higher species richness of graminoids and forbs (Figure 2C,D) as well as a higher density of larger trees than the forest plots. Riparian plot 1 (R1) contained a thick stand of alder ( *Alnus viridis* ) covering the western‐most fifth of the plot and similarly dense willows (*Salix* spp.) covering the eastern‐most fifth. Approximately half of Riparian plot 2 (R2) overlapped a meadow that was often covered by overflow in winter and early spring (Figure 2D).

### 2.2 Field Methods

National Park Service (NPS) staff monitored small mammal populations annually in four 100 × 100 m plots as part of a long‐term monitoring program that began in 1993, although not all plots were established until 1997. Autumn trapping took place during mid‐August for all years (1993–2022), when voles had not yet begun to experience winter mortality and were at their presumed peak population for the year. Early summer trapping was conducted during the second or third week of June in 1994–2002 and 2020–2022. Each of the four plots contained 100 Sherman live‐traps arranged in a square grid with 10 m spacing. Traps were protected by plastic covers to keep animals dry during rain and shaded from sun. We baited each trap with sunflower seeds and stocked them with two cotton nestlets, which animals could use to make nests for warmth. We checked each trap three times daily (06:00, 13:00, 20:00) for four consecutive days. We scanned each animal upon capture using a Passive Integrative Transponder (PIT) tag reader to establish capture status, marked or unmarked. We marked each unmarked animal with a unique 9–12 mm PIT tag (Biomark, Boise, ID) injected into their subcutaneous fat. We conducted all trapping and handling in accordance with the guidelines of the American Society of Mammalogy (Sikes 2016), and procedures were approved by the NPS institutional animal care and use committee (NPS IACUC, protocol identifier AK_DENA_Swanson_Vole.Shrew_2020.A1). See Flamme et al. (2018) for further details on plot set up and trapping procedures and Swanson (2025) for published dataset.

### 2.3 Covariates

#### 2.3.1 Climate

We obtained weather data from a station near Denali National Park Headquarters, located approximately 1.6 km from our study area. All snow‐related variables were measured daily during the snow year, defined as the period between the last day in a calendar year with snow depth = 0 cm (snow‐on date) and first day of the following spring when snow depth = 0 cm (snow‐off date). We selected variables for analysis based on the potential for thermal stress (due to late or little snowpack), limited mobility (due to snow compaction or ice layers resulting from temperatures above freezing), and food availability for voles (Table 1). We used melt and rain days (days with average daily temperature above 0°C and measurable precipitation, respectively) as proxies for icing and compaction in the snowpack. To specifically address events that would have potential to impact the bottom levels of the snowpack, this was limited to rain or melt that occurred with less than 30 cm of snow on the ground, correlating with the upper bounds maximum snow insulation (Reid et al. 2012). We also created two indices of autumn harshness: (1) total days per year with a daily minimum temperature below −17.8°C prior to the establishment of sufficient snow insulation (> 15 cm, similar to Schmidt et al. 2018 and following Pruitt 1957); and (2) a binary variable indicating > 5 days in a year with the above conditions. The threshold of −17.8°C was chosen to correspond with 0°F and be slightly above the highest measured lower lethal temperature for red‐backed voles of −19°C (Rosenmann et al. 1975). We also included spring precipitation (cumulative April and May precipitation), summer precipitation (cumulative precipitation between snow off date and August 1), and growing degree days (GDD) with a base of 5°C. The combination of GDD, cumulative summer precipitation, and mean snow depth has been shown to explain most of the variation in average annual maximum Normalized Difference Vegetation Index (NDVI), a proxy for primary productivity (Schmidt et al. 2018). We used a 3‐year moving average of these three variables to align with the short generation times in vole populations while also accommodating the initiation period for white spruce mast (Roland et al. 2014). For variables representing the growing season leading up to a trapping session, we used a cut‐off of 1 August to restrict weather events to only those that would have affected animals prior to monitoring. Collinearity of variables was assessed, and we found no values above 0.7 for covariates that were not linked in how they were calculated (such as potential melt and rain days); however, these correlated terms were never used in the same model as the use of one excluded the use of another.

**TABLE 1:** Complete list of variables used for linear models. Use category specifies whether a variable was used to model differences in density between autumn and the following early summer (S) or in autumn only models (A).

Variable name	Use	Description
*Environmental variables*		
AddSnowDepth	S, A	Additive snow depth calculated throughout the snow year
MnSnowDepth	S, A	Mean snow depth, calculated as additive snow depth/days in snow year
MeltDays	S, A	Number of days between snow on date and establishment of at least 30 cm of snow during which mean temperature exceeded 0°C
MeltDaysB	S, A	Binary version of MeltDays to represent threshold of 5 days of melt prior to establishment of > 30 cm of snow
RainDays	S, A	Number of days between snow on date and establishment of at least 30 cm of snow during which minimum temperature was below 0°C and precipitation was recorded
RainDaysB	S, A	Binary version of RainDays to represent threshold of 2 days of rain during prior to establishment of > 30 cm of snow
ColdDays	S, A	Number of days, beginning in August, for which minimum temperature was below −17°C and snow depth had yet to exceed the thermal threshold of 15 cm
ColdDaysB	S, A	Binary version of ColdDays to represent a threshold of 5 cold days prior to establishment of > 15 cm of snow
SeasonLength	S, A	Number of days in a given snow season relative to the average snow season found during the study period
SpringPrecip	S, A	Cumulative precipitation falling in April and May
SummerPrecip	A	Cumulative precipitation falling between snow off and August 1
PrevSummerPrecip	A	Cumulative precipitation falling between snow off and snow on during the previous summer
GDD	A	Growing degree days, metric of plant growth potential when average temperature exceeds 5°C through August 1
GDDPrev	A	Growing degree days, metric of plant growth potential when average temperature exceeds 5°C throughout the previous summer
Productivity	A	Combination of 3‐year retrogressive averages of mean snow depth, cumulative summer precipitation, and growing degree days to represent multi‐year increased primary productivity
Seeds	S, A	White spruce ( *Picea glauca* ) seed fall, measured in mean seeds captured in seed traps (see Roland et al. 2014 for protocol)
*Density dependent variables*		
PrevHiLo	S, A	Binary expression of autumn density estimate from the previous year, with 10 voles/ha as the threshold distinguishing high from low
AutumnEst	S, A	Bootstrapped distribution of densities from autumn of the previous year, constructed using the mean and standard error of the top‐ranking model for each plot
Cosine	S, A	Cosine wave with 2‐ or 3‐year period
Sine	S, A	Sine wave with 3‐year period (2‐year period resulted in zero values)

#### 2.3.2 Spruce Seeds

We were interested in evaluating the influence of spruce seed abundance on red‐backed voles, based on other species of vole ( *Clethrionomys gapperi* ) and mice ( *Peromyscus maniculatus* ) appearing to use spruce seeds as food sources (Elias et al. 2006; Falls et al. 2007). Seeds have been collected at 3 plots within 0.5 km of the riparian plots since 1992 (see Roland et al. 2014 for details on spruce seed collection). We took a mean of these measurements to create an index representing spruce seed abundance for our study area (see Table A1 for seedfall data). Seeds attributed to a given year were those that fell the previous autumn and would be available as a food resource throughout the preceding winter.

### 2.4 Analytical Methods

#### 2.4.1 Density Estimation

We used a two‐stage post hoc linear modeling approach to explore the relationship between seasonal density estimates and environmental factors. In the first stage, we estimated plot‐level densities for each season (autumn and early summer). Plots were assessed separately, despite some being close in proximity, due to limited overlap in individuals. A maximum of two individuals passed between nearby riparian plots each year, with the exception of 2017 (the highest capture year on record) in which 4 individuals passed between R1 and R2. We fit six different models to the data from each plot in each season, all of which included variation in density (*D*) by year, one of three options for detection probability—a null model, variation by year, and a two‐class mixture representing individual heterogeneity, essentially classifying an animal as either trap‐happy or trap‐shy—and one of two options representing home‐range size, *σ*, which was either held constant or varied by year. We constrained the potential area from which individuals might be exposed to traps to be 4*σ*, in our case 120 m (see Efford 2020). These models were then compared using a small sample corrected Akaike Information Criterion (AICc) values to select the most‐parsimonious structure (Burnham and Anderson 2002). We fit all models using the SECR package (version 4.3.1) in program R, version 4.0.2 (Efford 2020; Efford and Fewster 2013; R Core Team 2023).

#### 2.4.2 Population Cycling

As part of the first stage, we also conducted an exploratory analysis of population cyclicity. Given that we expected a cyclic pattern in the density estimates (i.e., Schmidt et al. 2018), we plotted the autocorrelation function for our series of density estimates from each plot using the acf function in R (R Core Team 2023) and used the results to inform the period of sine and cosine terms representing cyclicity in models constructed during the second stage. We also explored the potential for cyclic patterns in the data by fitting a second‐order log‐linear model to the series of density estimates from each plot to assess direct and delayed density dependence (Bjornstad et al. 1995; Fauteux et al. 2015; Ims et al. 2008; Royama 1992; Stenseth 1999). This model can be written as:

(1) $$x_{t}=\beta_{0}+\left(1+\beta_{1}\right)x_{t-1}+\beta_{2}x_{t-2}$$

where $x_{t}$ represents log‐transformed vole density at year t. *β*_0_ is the intercept, and the first‐order term (1 + *β*_1_) represents the coefficient for direct density dependence in year *t*−1, which could take the form of population self‐regulation or a predator response. The second‐order term (*β*_2_) represents the coefficient for delayed density dependence in year *t*−2, such as a less rapid predator response or increased pathogens in the population. The size of these coefficients indicated the influence of past years' densities on current year density. We displayed these terms for each plot on a phase plane diagram (Fauteux et al. 2015) which distinguishes between cyclic and non‐cyclic populations, and further predicts period length for those that fall in the cyclic realm (Royama 1992; Ims et al. 2008). Due to the number of years in which early summer trapping did not occur, mid‐ we only conducted these analyses using our autumn density estimates.

#### 2.4.3 Factors Affecting Density

In the second stage of our analysis, we used a post hoc linear modeling approach to explore the relationship between environmental factors and the seasonal density estimates from stage one. We chose this approach, rather than directly including covariates in stage one, to improve model convergence and facilitate efficient model selection (e.g., Fauteux et al. 2015, 2021; Bogdziewicz et al. 2016). To account for the uncertainty surrounding the annual density estimates in each plot provided by the SECR analysis, we constructed a bootstrapped dataset in which each year‐plot included 1000 densities selected randomly using the empirical means and standard errors of the estimates from stage one. We truncated the distributions at 0 so that negative densities would not be included. This ensured that density estimates were not treated as points in the post hoc analysis, but rather a distribution. At each step in the model building, we used the bootstrapped density values as response variables in a suite of linear models. For each of the 1000 iterations, we ranked the models in a given time‐series based on AICc values to determine best fit (Burnham and Anderson 2002), and created a final summary table including average model rank, standard deviation of model rank, and average ΔAICc.

In the first step of model building, we explored various forms of population cycling and density dependence. Based on the findings from the exploratory analysis above, we evaluated models that included sine and cosine terms with 2‐ or 3‐year periods, as well as a model with no density dependent effects. For our models of autumn density, we also included a random effect for year representing unexplained variation among years (inclusion of this term in our early summer models was not supported by AICc model selection results). For early summer, we also evaluated models that included the density of the previous autumn and models that included a binary covariate representing whether the previous autumn density was high (≥ 10 voles/ha) or low (< 10 voles/ha) based on preliminary data visualization. This included data from spring of 2020, which would have been paired with incomplete data from autumn of 2019, during which only riparian plots were sampled due to flooding of Rock Creek blocking access to forest plots. Given that we had data for riparian plots, we used linear models to quantify the relationship between densities on forest plots and riparian plots in years of high density (2002, 2005, 2008, 2009, 2011, 2014, 2017, 2021, and 2022; note 2019 excluded due to missing data). Plot R2 showed the highest correlation with forest plots and was thus used to estimate the lag year covariate for forest plots in 2019.

Once we identified the most‐parsimonious form of cycling and density dependence, we iteratively added environmental variables relevant to each season (Table 1). This included the group of three variables that had been previously used to account for accumulated changes in NDVI, which served as a proxy for primary productivity (See Productivity in Table 1) (Schmidt et al. 2018). We selected the variable that ranked highest when added to the top density dependent model from the first step, then added all remaining environmental variables to that model. We continued this iterative addition of remaining variables until the top model ceased to have an average ΔAICc of 2 points over the next best model, or additional variables ceased to improve model fit (see Table B1). Prior to model building, we normalized all density estimates and centered and scaled our predictors to aid in model convergence and comparisons of effect sizes.

## 3 Results

### 3.1 Trapping Efforts

From 1993 and 2022, we captured a total of 3417 individual northern red‐backed voles a collective 11,428 times across the four plots and two seasons (Table B1). Of these, we had 1470 captures of 381 unique individuals during early summer trapping (range 4–324 captures, 3–82 individuals) and 9958 captures of 3144 unique individuals during autumn trapping (range 50–1448 captures, 17–367 individuals). Only 108 animals were captured in both early summer and fall. Animals varied in response to initial capture, with just over 41% being caught only once and others returning to traps on multiple occasions.

### 3.2 Autumn Density

**FIGURE 3:**
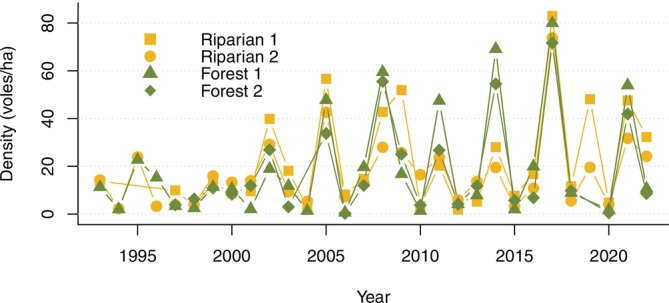
Autumn density estimates (voles/ha) for northern red‐backed voles ( *Clethrionomys rutilus* ) on four plots in the Rock Creek watershed of Denali National Park and Preserve. All plots were 100 m^2^, included 100 traps, and were monitored for four days during mid‐August.

For all four plots, the model including individual heterogeneity in capture probability (*g*_0_) and variation in home range size (*σ*) by year was the most parsimonious explanation of the capture data, with an AICc weight of 1 for all plots. Red‐backed vole density estimates for autumn displayed similar timing of highs and lows across plots, but with differing amplitudes (Figure 3). Density estimates varied dramatically during the sampling period, with autumn density estimates ranging from years with no detections at all (F2, 2004) to ~80 voles/ha on F1 in 2017.

**FIGURE 4:**
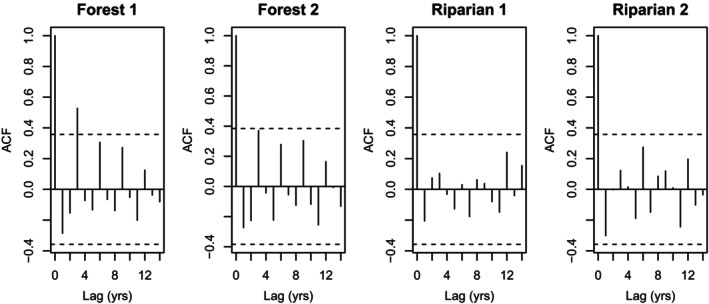
Autocorrelation plots (ACF = autocorrelation function) for northern red‐backed vole ( *Clethrionomys rutilus* ) density estimates found on each of the four plots in the Rock Creek watershed of Denali National Park and Preserve. Dotted lines represent a 95% confidence interval, and lines that extend beyond this interval represent significant autocorrelation (cyclicity) for a given number of years.

**FIGURE 5:**
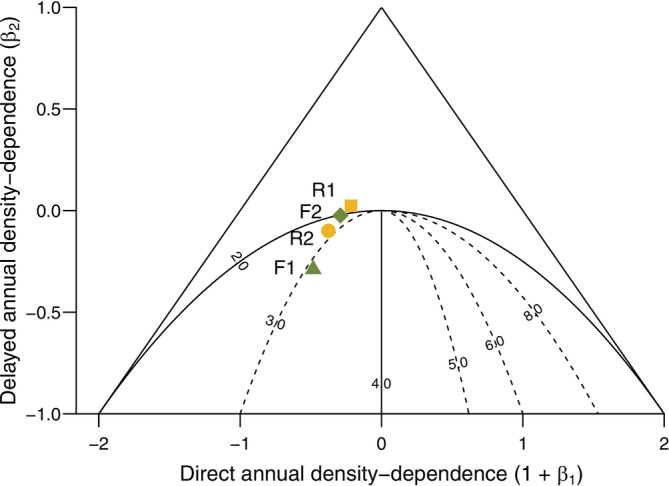
Phase plane diagram displaying estimated strengths of direct and delayed density dependence for four plots in the Rock Creek watershed measured during autumn. Values represent beta coefficients from a log‐linear autoregressive model ($x_{t}=\beta_{0}+\left(1+\beta_{1}\right)x_{t-1}+\beta_{2}x_{t-2}$) over the 30‐year time series from 1993 to 2022. Points falling above the semicircle represent a lack of density‐dependence, while those falling within the semicircle represent cyclicity on a period specified by section. Values of the dotted lines correspond to the cyclicity (years) identified by the model. The triangle signifies the range of possible values of beta coefficient combinations for which populations are sustained over time (Stenseth 1999). Period strength increases through a decrease in delayed density‐dependence ($\beta_{2}$) while period length increases with higher direct density‐dependence $\left(1+\beta_{1}\right)$ if the corresponding delayed coefficient is in the cyclic region.

Only one plot (F1) exhibited a significant cyclical pattern based on post hoc autocorrelation analysis (Figure 4). The three‐year periodicity of this plot was also evident in the phase plane diagram (Figure 5). A similar, but non‐significant, pattern in autocorrelation was visible for F2, which registers as nearly cyclic with a three‐year period on the phase diagram. Plot R1 showed little evidence of cyclicity, but R2 appeared to be weakly cyclic on the phase plane diagram (Figures 4 and 5).

### 3.3 Early Summer Density

Similar to autumn, for all four plots in early summer, the model that included heterogeneity in capture probability was the most parsimonious. Unlike our autumn models, however, we had insufficient data to fit models including variation in home‐range size. Early summer density estimates ranged from 0.3 to 5.8 voles/ha, showing less pronounced variation than those for autumn sessions.

### 3.4 Factors Affecting Density

Our top model of autumn density included a sine wave with a period of 3, white spruce seed abundance, and a binary covariate for autumn harshness (mean $R_{\text{marginal}}^{2}$=0.36, mean ΔAICc 0.087, see Table C1 for full model selection table). Increased white spruce seed availability in a given autumn season was associated with higher vole density nearly a year later (*β*_seeds_ = 0.50, 95% CI [0.20, 0.80]), whereas binary autumn harshness was associated with decreased density in the following autumn (*β*_ColdDaysB_ = −0.63, 95% CI [−1.20, −0.04]).

**FIGURE 6:**
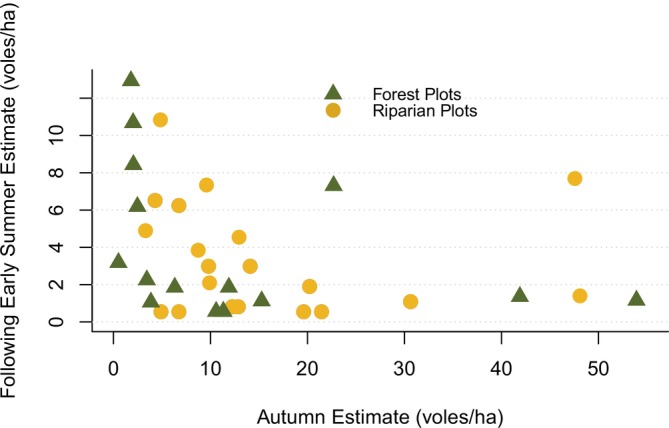
Northern red‐backed vole ( *Clethrionomys rutilus* ) density estimates for the autumn and following early summer, measured between 1993‐2002 and 2019‐2022 in Denali National Park and Preserve, Alaska. All autumn estimates were taken from sampling that occurred during the second week of August, while early summer estimates for most years came from the third week of June. Early summer estimates were taken after the first litter was born, causing some years to show higher densities in early summer than had been present in autumn.

We found no evidence for any habitat or weather factors affecting early summer density. We found a strong inverse relationship between autumn densities and the following early summer, in which high autumn population densities were generally followed by lower early summer densities (Figure 6). For the post hoc linear modeling, this relationship was best represented using a binary classification of autumn density as high or low, with 10 voles/ha serving as the threshold between categories (*β*_PrevHiLo_ = −0.97, 95% CI [−1.73, −0.20]) (Table D1). White spruce seeds that fell the previous autumn were ranked highest against all other environmental variables, but did not show substantial improvement in fit over the density dependent model.

## 4 Discussion

We evaluated long‐term patterns in seasonal density of a cyclical population of small mammals in the boreal ecosystem of Alaska, leveraging three decades of co‐located monitoring efforts to assess relationships between northern red‐backed voles and their environment. We found that autumn densities were positively affected by spruce seed abundance during the previous winter, a linkage that had previously not been observed for northern red‐backed voles, and negatively affected by harsh weather conditions in the early winter. We also found that the strength of population cycles appears to be dampening compared to the last analysis of this population (Schmidt et al. 2018), with more variation in cyclic period but continued high amplitudes, unlike patterns seen in Europe (Cornulier et al. 2013). This, coupled with our finding that early summer densities were more strongly related to densities the previous autumn than with environmental conditions, indicates that density dependence remains an important factor in vole population dynamics.

Our results suggested a less consistent cycle compared to earlier work on this population (Schmidt et al. 2018). The pattern we observed was similar to those presented in a classic paper describing chaos in populations with non‐overlapping generations (May 1974), in which a population occasionally displayed a 3‐year period bracketed by more erratic behavior. Although vole generations do overlap temporally, mortality during winter is sufficiently high that parallels can still be drawn. Chaotic patterns have also been explained by mustelid predation, despite prey retaining a statistically significant periodic element (Hanski et al. 1993). We lack data on mustelids in our area of study, but continued long‐term monitoring in autumn may shed light on the issue of periodicity.

With amplitudes remaining consistent or even increasing over time, our results differed from instances of cyclic collapse observed in Europe. These collapses, characterized by substantial decreases in amplitudes beginning in the 1980s and 1990s, are generally characterized by populations eventually reaching a more stable but lower equilibrium (Cornulier et al. 2013; Ims et al. 2008). Conversely, our study saw an increase in amplitude during this time (Figure 3). The increase in amplitude seen during the mid‐ to late‐2000s and early 2010s was previously found to be related to increases in primary productivity (Schmidt et al. 2018), but we did not find a similar relationship. We suspect this was due in part to our analyzing all 4 plots separately, reducing our power to detect landscape‐scale covariate relationships in order to identify localized patterns in population density. Analyzing the plots separately did enable us to identify small‐scale environmental influences on red‐backed voles. No single plot exhibited consistently higher densities than others (Figure 3), and the peaks in densities among sampling plots were not always synchronized. Forest plots appeared to exhibit more cyclic fluctuations than the riparian plots, suggesting that while some factors may have affected all plots equally, others had more varied effects despite being less than 400 m apart. These plot‐level differences could also be due to differing species dynamics, given that riparian plots are also inhabited by tundra voles ( *Microtus oeconomus* ), which are larger, more herbivorous (Baltensperger et al. 2015), and occasionally have equal or even greater population densities than northern red‐backed voles, at which time they are equally important as both herbivores and prey. Tundra voles primarily occupy an area of R2 that exhibits ephemeral streams and a comparatively grassier habitat, which may also explain the generally lower population densities of northern red‐backed voles on R2. The presence of a separate prey species could allow predators to prey‐switch when their preferred species became less available, leading to weaker density‐dependent cyclicity if the cycle was partially predator driven (Hansson and Henttonen 1985). However, this diversity could allow the system to support more predators, given that they have slightly different habitat diet preferences, and would thus respond to different environmental triggers than red‐backed voles. A more in‐depth assessment of interspecific dynamics, specifically habitat preferences and density patterns of all species present on the landscape, could shed light on this aspect.

One novel finding from our study was the positive relationship between white spruce mast and density, which has not been identified in past studies of northern red‐backed voles (Krebs et al. 2010). This relationship suggests that white spruce seeds may represent an important winter food source for red‐backed voles when available. Studies of vole stomach contents have indicated consumption of white spruce seeds, and voles presented with them in laboratory trials readily eaten them, although they tended to lose weight if their diets consisted entirely of spruce seeds (Grodzinski 1971). However, if spruce seeds were supplemented with lichen, animals maintained body weight, suggesting that they might be a helpful addition to the overwinter food supply alongside more consistently available sources (Grodzinski 1971). A similar trend was observed in southern Norway, where spruce seed mast from the previous fall was identified as a mechanism for increased winter survival of the bank vole *(Clethrionomys glareolus)* (Selas 2002). Although spruce seeds may represent a direct food source, previous studies have found that weather events up to 3 years prior to seed fall can affect the magnitude of mast, such as low snowfall in the winter prior to seed fall and a cool, wet summer in the months leading up to seeds becoming available (Juday 2003; Roland et al. 2014), which could also affect vole populations directly. Weather events related to spruce mast could also affect production of other vole food sources, such as berries, which appear dependent on multi‐annual weather patterns (Krebs et al. 2009). Further exploration of this potential link between vole populations and spruce seed abundance, such as a thorough exploration of vole diet, is important given changes occurring in our study area, specifically an outbreak of spruce beetles that moved northward into the area as of 2022 (Stehn and Syrotchen 2022). This type of infestation has had mixed effects on vole populations in other regions (Lance et al. 2006; McDonough and Rexstad 2005), and could dampen the dramatic amplitude fluctuations seen in our study area if voles are truly depending on spruce seeds as a food source.

We also found that late snow onset coupled with cold weather was associated with low vole populations the following fall. This relationship is relevant given predictions for progressively later snow onset based on climate projections (Lader et al. 2020). Shortened snow seasons have been linked to cyclic collapse of voles in Europe (Ims et al. 2008), potentially due to shifts between specialist and generalist predation that usually accompany seasonal change (Tyson and Lutscher 2016). Coupled with predictions for decreased snow season length as a result of climate change (Lader et al. 2020), this mechanism suggests a tipping point which could lead to dramatic changes to small mammal populations. Similarly, rain‐on‐snow events could become a prominent factor affecting winter population dynamics because of climate change. Although not in our top model, preliminary examination of autumn densities and early‐snow‐season rain‐on‐snow days showed a limiting relationship, in which high rain‐on‐snow occurrences appeared to prevent densities the following year from spiking. This aligns with findings of other studies (Fauteux et al. 2021; Soininen et al. 2015) and can be explained by the potential for higher density snow near the base of the snowpack, with accompanying effects on mobility, food availability, and decreased insulative value. The timing of such events could be crucial, as ice crusts higher in the snowpack may serve as a helpful barrier to plunging predators (Berteaux et al. 2017). Later season rain‐on‐snow events were not examined but could prove helpful for predicting patterns in vole density resulting from more variable winter weather.

Our only predictor of early summer density was density in the previous autumn. This pattern is illustrated by F1, which showed both an uncharacteristically low autumn density of less than 2 voles/ha in 2020 followed by an early summer with around 10 voles/ha, and an extreme high of over 50 voles/ha in autumn 2021 followed by a crash to < 2 voles/ha the next early summer. Although voles are likely not reproductive during winter (Stevenson et al. 2009), our sampling timing was such that the first litter born to overwintered animals was out of the nest, causing the apparent increases over winter months. Almost no years with elevated densities in the autumn remained high, but not all years with low autumn population were followed by high early summer densities, likely a product of the many other environmental or internal factors that could limit populations from spiking. This suggests a sort of density dependence, but with an unknown cause. Increased mortality after a peak could be a result of food shortage over the winter months due to the population being at or near carrying capacity, similar to patterns found in other vole species (Huitu et al. 2003; Johnsen et al. 2017). However, it could also represent a predator response, wherein a specialist predator showed a strong but delayed population increase in reaction to heightened prey availability. Future work incorporating data on vole predator populations and their diets could aid in investigating the potential implications of a predator response to prey availability. Lastly, this result differs from our observations from the full autumn dataset in that overwinter weather effects like fall harshness do not appear to be influential. This could be a result of either small sample size or that most paired autumn/early summer data were collected in the 1990s, when fall harshness was generally lower and potentially less impactful. Continued monitoring of early summer vole populations, which is not included in the current protocol, could help to resolve both issues, potentially providing high enough sample size for more complex models and increasing the chances of capturing changing climate effects.

During the last three decades, the vole populations at the study site in Denali National Park and Preserve have shown cyclicity, but with some variation in both period and amplitude. Here we identified cyclic patterns and environmental factors affecting densities, highlighting the importance of long‐term monitoring for understanding the complex dynamics of small mammal populations. Although we did not identify a strong relationship between snow conditions and density, continued monitoring of snow characteristics may facilitate a better understanding of how winter conditions influence small mammal populations. Including research on vole predators could illuminate many of the existing uncertainties highlighted by this study, including the mechanism of winter declines and cyclicity. This could also help discern the potential ramifications of changes in snow season and assess how alterations in vole populations over time could affect higher trophic levels within interior Alaska. As short‐lived mammals with high reproductive rates, red‐backed vole populations may serve as an early warning sign of a changing ecosystem.

## Author Contributions

**Sarah Swanson:** conceptualization (equal), formal analysis (lead), writing – original draft (lead). **Melanie Flamme:** conceptualization (equal), funding acquisition (lead), project administration (lead). **Josh Schmidt:** conceptualization (supporting), formal analysis (supporting), writing – review and editing (equal). **Shawn Crimmins:** formal analysis (supporting), writing – review and editing (equal). **Carl Roland:** conceptualization (supporting), resources (supporting), writing – review and editing (supporting). **Knut Kielland:** conceptualization (supporting), formal analysis (supporting), supervision (lead), writing – review and editing (equal).

## Conflicts of Interest

The authors declare no conflicts of interest.

## Data Availability

The data and code that support the findings of this study are publicly available in the NPS IRMA datastore at https://doi.org/10.57830/2315265, reference number 2315265.

## Appendix A

**TABLE A1:** White spruce ( *Picea glauca* ) seed data collected near Rock Creek small mammal plots in Denali National Park, Alaska, throughout sampling period, measured in mean seeds per m^2^. Seed data collection protocol is detailed in Roland et al. (2014).

Year	Spruce seeds (m^2^)
1993	4
1994	51
1995	2
1996	21
1997	51
1998	471
1999	18
2000	90
2001	13
2002	199
2003	7
2004	10
2005	57
2006	6
2007	1
2008	299
2009	19
2010	289
2011	15
2012	16
2013	294
2014	132
2015	34
2016	689
2017	17
2018	96
2019	79
2020	1110
2021	51

## Appendix B

**TABLE B1:** Northern red‐backed vole ( *Clethrionomys rutilus* ) trapping success by year, plot, and season, with number of unique individuals noted first followed by number of total captures in parentheses. Trapping occurred in the Rock Creek drainage in Denali National Park, Alaska. Forest plots are denoted by ‘F’ and riparian plots with ‘R’. Plots without numbers listed were not trapped in the given year.

Year	Fall trapping				Early summer trapping			
	F1	F2	R1	R2	F1	F2	R1	R2
1993	27 (117)		23 (61)	32 (111)	18 (60)		16 (69)	11 (35)
1994	10 (42)			7 (27)	1 (1)			2 (3)
1995	39 (162)			33 (82)	19 (86)			24 (81)
1996	27 (72)			11 (37)	13 (49)			7 (35)
1997	5 (13)	2 (2)	18 (55)	8 (26)	2 (2)	1 (1)	9 (32)	2 (4)
1998	10 (30)	18 (63)	13 (40)	16 (35)	4 (17)	4 (10)	6 (25)	2 (4)
1999	24 (96)	24 (100)	21 (63)	20 (73)	11 (46)	7 (25)	14 (56)	11 (26)
2000	13 (75)	13 (40)	19 (109)	21 (90)	1 (1)		13 (45)	3 (11)
2001	10 (36)	17 (75)	22 (78)	23 (95)	1 (1)		11 (35)	3 (18)
2002	37 (98)	43 (125)	51 (153)	37 (90)	15 (42)	7 (16)	21 (70)	11 (51)
2003	13 (38)	10 (30)	24 (83)	16 (64)				
2004	6 (31)	0	8 (21)	6 (11)				
2005	56 (185)	43 (156)	74 (222)	51 (98)				
2006	2 (7)	1 (1)	16 (31)	7 (31)				
2007	39 (67)	19 (31)	32 (61)	15 (61)				
2008	100 (321)	88 (310)	68 (217)	48 (156)				
2009	29 (103)	33 (83)	66 (158)	41 (126)				
2010	2 (11)	3 (6)	8 (16)	11 (20)				
2011	46 (97)	41 (93)	40 (81)	47 (86)				
2012	15 (35)	9 (20)	4 (9)	11 (20)				
2013	29 (57)	26 (59)	13 (44)	18 (44)				
2014	75 (209)	82 (244)	44 (121)	29 (91)				
2015	11 (31)	6 (11)	24 (71)	15 (36)				
2016	28 (93)	19 (74)	31 (100)	24 (63)				
2017	92 (385)	85 (331)	107 (438)	88 (294)				
2018	16 (29)	14 (37)	12 (28)	10 (17)				
2019			52 (144)	35 (111)				
2020	3 (3)	4 (17)	17 (52)	11 (49)	3 (8)	2 (6)	4 (12)	2 (7)
2021	78 (280)	60 (191)	71 (248)	50 (153)	23 (90)	12 (64)	31 (89)	23 (81)
2022	22 (99)	14 (39)	33 (84)	27 (69)	2 (12)	5 (19)	21 (113)	4 (11)

## Appendix C

**TABLE C1:** Model ranking structure for fall amplitude analysis of northern red‐backed voles ( *Clethrionomys rutilus* ) in the Rock Creek drainage of Denali National Park, Alaska. Models were fit using the lmer function in the lme4 package in Program R (R Core Team 2023). Values in this table represent summary statistics from 1000 iterations of each model, in which response variables were density estimates pulled from a bootstrapped dataset. This table shows the mean and standard deviation (SD) for model rank, mean and standard deviation of model weight, and mean and standard deviation of Delta AIC (Akaike Information Criterion).

	Model	Mean rank	Rank SD	Mean Wt	Wt SD	Mean ΔAIC	ΔAIC SD
*Density dependent (DD) variables*							
1	Dt ~ sine3 + (1|year)	1.122	0.3595	0.3602	0.0903	0.0679	0.2678
2	Dt ~ cos2 + (1|year)	2.676	1.2404	0.1641	0.0723	1.7784	1.2438
3	Dt ~ cos3 + sine3 + (1|year)	3.38	1.2341	0.1191	0.0293	2.2756	0.4258
4	Dt ~ 1 + (1|year)	4.048	1.0053	0.0998	0.0258	2.6371	0.8298
5	Dt ~ PrevHiLo + (1|year)	4.912	1.7763	0.0852	0.0582	3.2421	1.4213
6	Dt ~ Dt‐1 + sine3 + (1|year)	5.719	1.4100	0.0563	0.0313	3.9533	1.0572
7	Dt ~ cos3 + (1|year)	7.417	1.4857	0.0331	0.0109	4.8802	0.9739
8	Dt ~ Dt‐1 + (1|year)	8.539	1.2618	0.0249	0.0193	5.7573	1.5593
9	Dt ~ Dt‐1 + cos2 + (1|year)	8.577	1.2968	0.0241	0.0151	5.7332	1.5183
10	Dt ~ Dt‐1 + cos3 + sine3 + (1|year)	8.74	1.3536	0.0226	0.0150	5.8601	1.2685
11	Dt ~ Dt‐1 + cos3 + (1|year)	10.87	0.5073	0.0104	0.0097	7.6633	1.7975
*Environmental variable models*							
1	Dt ~ sine3 + seedscale + ColdDaysB + (1|year)	1.233	0.5301	0.1470	0.0314	0.0872	0.2437
2	Dt ~ sine3 + seedscale + ColdDaysB + RainDaysB + (1|year)	3.05	1.4005	0.0891	0.0350	1.1834	0.6538
3	Dt ~ sine3 + seedscale + (1|year)	3.206	1.8759	0.0831	0.0255	1.2847	0.9405
4	Dt ~ sine3 + seedscale + RainDaysB + (1|year)	4.251	2.1087	0.0680	0.0230	1.6936	0.9206
5	Dt ~ sine3 + seedscale + ColdDaysB + MeltDaysB + (1|year)	5.225	1.8493	0.0522	0.0158	2.1929	0.5092
6	Dt ~ sine3 + seedscale + ColdDaysB + SumPrecipPrevS + (1|year)	7.938	2.8316	0.0371	0.0117	2.8804	0.6178
7	Dt ~ sine3 + seedscale + SumPrecipPrevS + (1|year)	9.202	4.4939	0.0368	0.0158	2.9842	1.1242
8	Dt ~ sine3 + seedscale + ColdDaysB + RainDays + (1|year)	9.284	4.5942	0.0347	0.0129	3.0524	0.6030
9	Dt ~ sine3 + seedscale + ColdDaysB + AvgDepthS + (1|year)	9.672	2.8363	0.0291	0.0069	3.3352	0.3669
10	Dt ~ sine3 + seedscale + MeltDaysB + (1|year)	10.823	4.9736	0.0312	0.0136	3.3177	1.1050
11	Dt ~ sine3 + seedscale + ColdDaysB + IntSnowDepthS + (1|year)	11.433	3.0563	0.0269	0.0059	3.4847	0.3264
12	Dt ~ sine3 + seedscale + ColdDaysB + LengthS + (1|year)	13.583	4.6459	0.0247	0.0067	3.6771	0.3908
13	Dt ~ sine3 + seedscale + ColdDaysB + SumPrecipS + (1|year)	13.716	4.2755	0.0239	0.0062	3.7329	0.3806
14	Dt ~ sine3 + seedscale + ColdDaysB + SprPrecipS + (1|year)	15.471	3.9351	0.0215	0.0044	3.9298	0.2645
15	Dt ~ sine3 + RainDaysB + (1|year)	16.072	7.5274	0.0228	0.0107	3.9850	1.1774
16	Dt ~ sine3 + seedscale + ColdDaysB + GDDPrevS + (1|year)	17.71	4.3378	0.0199	0.0040	4.0832	0.2692
17	Dt ~ sine3 + seedscale + RainDays + (1|year)	18.147	4.3304	0.0172	0.0047	4.4110	0.8889
18	Dt ~ sine3 + seedscale + ColdDaysB + MeltDays + (1|year)	18.472	4.5008	0.0195	0.0040	4.1193	0.2752
19	Dt ~ sine3 + seedscale + ColdDaysB + GDDS + (1|year)	19.66	4.6802	0.0189	0.0039	4.1872	0.2683
20	Dt ~ sine3 + seedscale + SprPrecipS + (1|year)	20.317	3.7022	0.0150	0.0039	4.6733	0.8225
21	Dt ~ sine3 + seedscale + IntSnowDepthS + (1|year)	20.772	3.7172	0.0144	0.0041	4.7792	0.9013
22	Dt ~ seedscale + (1|year)	21.129	8.2615	0.0168	0.0104	4.7449	1.5001
23	Dt ~ sine3 + seedscale + AvgDepthS + (1|year)	21.486	3.8829	0.0142	0.0042	4.8075	0.9116
24	Dt ~ sine3 + seedscale + ColdDaysS + (1|year)	22.775	3.1516	0.0137	0.0033	4.8519	0.7802
25	Dt ~ sine3 + ColdDaysB + (1|year)	22.9	6.0087	0.0147	0.0050	4.7649	0.7688
26	Dt ~ sine3 + seedscale + SumPrecipS + (1|year)	23.159	4.2998	0.0134	0.0043	4.9344	0.9549
27	Dt ~ sine3 + seedscale + GDDPrevS + (1|year)	25.659	3.2129	0.0122	0.0037	5.1158	0.9395
28	Dt ~ sine3 + seedscale + MeltDays + (1|year)	26.608	3.2703	0.0119	0.0036	5.1694	0.9274
29	Dt ~ sine3 + (1|year)	27.602	4.6677	0.0107	0.0042	5.4364	1.0819
30	Dt ~ sine3 + seedscale + LengthS + (1|year)	27.98	2.7231	0.0115	0.0035	5.2348	0.9393
31	Dt ~ sine3 + seedscale + GDDS + (1|year)	28.06	3.8080	0.0116	0.0040	5.2415	0.9806
32	Dt ~ sine3 + RainDays + (1|year)	32.017	1.9831	0.0051	0.0022	6.9379	1.1195
33	Dt ~ sine3 + MeltDaysB + (1|year)	33.57	0.7986	0.0036	0.0014	7.6239	1.0834
34	Dt ~ 1 + (1|year)	35.923	4.4000	0.0034	0.0024	8.0056	1.5809
35	Dt ~ sine3 + seedscale + ColdDaysB + SumPrecip3retavgS + AvgDepth3retavgS + GDD3retavgS + (1|year)	37.085	5.0374	0.0028	0.0012	8.1540	0.8325
36	Dt ~ sine3 + AvgDepthS + (1|year)	38.109	1.7076	0.0021	0.0009	8.6689	1.1170
37	Dt ~ sine3 + SumPrecipPrevS + (1|year)	38.151	2.5967	0.0023	0.0010	8.5643	1.1765
38	Dt ~ sine3 + SprPrecipS + (1|year)	38.395	2.3273	0.0022	0.0008	8.6258	1.0334
39	Dt ~ sine3 + MeltDays + (1|year)	38.438	2.8157	0.0022	0.0008	8.5713	1.0115
40	Dt ~ sine3 + SumPrecipS + (1|year)	39.232	2.7553	0.0021	0.0009	8.6846	1.1444
41	Dt ~ sine3 + IntSnowDepthS + (1|year)	39.314	1.6960	0.0021	0.0009	8.6940	1.1140
42	Dt ~ sine3 + GDDPrevS + (1|year)	39.795	2.9543	0.0021	0.0009	8.7214	1.1298
43	Dt ~ sine3 + LengthS + (1|year)	42.074	2.3345	0.0019	0.0008	8.9002	1.1135
44	Dt ~ sine3 + ColdDaysS + (1|year)	42.828	2.0225	0.0018	0.0007	8.9585	1.0307
45	Dt ~ sine3 + GDDS + (1|year)	43.589	2.1519	0.0018	0.0009	9.0399	1.1735
46	Dt ~ sine3 + seedscale + SumPrecip3retavgS + AvgDepth3retavgS + GDD3retavgS + (1|year)	45.885	0.7323	0.0008	0.0004	10.6187	1.2658
47	Dt ~ sine3 + SumPrecip3retavgS + AvgDepth3retavgS + GDD3retavgS + (1|year)	47	0.0000	0.0001	0.0000	15.6203	1.2496

## Appendix D

**TABLE D1:** Model ranking structure for early summer analysis of northern red‐backed voles ( *Clethrionomys rutilus* ) in the Rock Creek drainage of Denali National Park, Alaska. Models were fit using the lm function in the Program R base stats package (R Core Team 2023). Values in this table represent summary statistics from 1000 iterations of each model, in which response variables were density estimates pulled from a bootstrapped dataset. This table shows the mean and standard deviation (SD) for model rank, mean and standard deviation of model weight, and mean and standard deviation of Delta AIC (Akaike information criterion).

	Model	Mean rank	Rank SD	Mean Wt	Wt SD	Mean delta AIC	Delta AIC SD
*Density dependent variables*							
1	Dt ~ PrevHiLo	1.486	1.0941	0.4280	0.2271	0.3255	0.7924
2	Dt ~ Dt‐1	2.947	1.4249	0.1232	0.0757	2.8473	1.9562
3	Dt ~ Dt‐1 + sine3	4.434	2.2691	0.0936	0.0808	3.6433	2.1920
4	Dt ~ cos2	5.054	2.4834	0.0794	0.0803	4.2601	2.5765
5	Dt ~ 1	5.334	2.1783	0.0597	0.0502	4.7463	2.6143
6	Dt ~ Dt‐1 + cos2	5.823	2.1749	0.0571	0.0371	4.4105	1.9785
7	Dt ~ Dt‐1 + cos3	6.432	2.1854	0.0500	0.0431	4.8160	2.1835
8	Dt ~ sine3	7.813	2.2066	0.0327	0.0346	6.1611	2.7885
9	Dt ~ Dt‐1 + sine3 + cos3	8.04	2.3698	0.0332	0.0329	5.7940	2.3333
10	Dt ~ cos3	8.292	1.9709	0.0290	0.0302	6.4155	2.8689
11	Dt ~ cos3 + sine3	10.345	1.2624	0.0139	0.0151	7.9941	2.9858
*Density dependent variables + environmental variables*							
1	Dt ~ PrevHiLo	2.712	1.2492	0.1153	0.0294	1.3227	1.2092
2	Dt ~ PrevHiLo + ColdDays	3.582	2.8955	0.1395	0.0961	1.3142	1.5510
3	Dt ~ PrevHiLo + seedscale	3.638	2.6423	0.1276	0.0877	1.4304	1.4587
4	Dt ~ PrevHiLo + SprPrecip	5.354	2.9919	0.0815	0.0524	2.2415	1.4640
5	Dt ~ PrevHiLo + RainDaysB	5.495	3.3489	0.0907	0.0700	2.1216	1.4194
6	Dt ~ PrevHiLo + RainDays	6.985	3.3815	0.0679	0.0513	2.6238	1.3400
7	Dt ~ PrevHiLo + SumPrecipPrev	9.475	3.6264	0.0556	0.0695	3.1570	1.3753
8	Dt ~ PrevHiLo + MeltDays	9.519	3.0034	0.0456	0.0187	3.2227	1.1869
9	Dt ~ PrevHiLo + MeltDaysB	9.654	2.9952	0.0438	0.0169	3.2918	1.1757
10	Dt ~ PrevHiLo + Length	9.735	3.0991	0.0431	0.0190	3.3481	1.2432
11	Dt ~ PrevHiLo + GDDPrev	9.933	3.3487	0.0427	0.0195	3.3899	1.2980
12	Dt ~ PrevHiLo + ColdDaysB	10.098	3.1794	0.0400	0.0127	3.4633	1.1474
13	Dt ~ PrevHiLo + IntSnowDepth	10.489	2.9439	0.0401	0.0161	3.4968	1.3161
14	Dt ~ PrevHiLo + AvgDepth	10.62	2.8375	0.0397	0.0152	3.5100	1.3016
15	Dt ~ 1	12.711	4.2890	0.0269	0.0369	5.7435	2.9461
